# Incubation period of typhoidal salmonellosis: a systematic review and meta-analysis of outbreaks and experimental studies occurring over the last century

**DOI:** 10.1186/s12879-018-3391-3

**Published:** 2018-09-27

**Authors:** Adedoyin Awofisayo-Okuyelu, Noel McCarthy, Ifunanya Mgbakor, Ian Hall

**Affiliations:** 1 NIHR Health Protection Research Unit in Gastrointestinal Infection, University of Liverpool, Liverpool, UK; 2 Department of Zoology, University of Oxford, Oxford, UK; 3 Warwick Medical School, University of Warwick, Warwick, UK; 4 Epidemiology, Strategic Information and Health Systems Strengthening Branch, Nigeria Office, Lagos, Nigeria; 5 School of Mathematics, University of Manchester, Manchester, UK

**Keywords:** Incubation period, Salmonella Typhi, Systematic review, Observational studies, Experimental studies

## Abstract

**Background:**

*Salmonella* Typhi is a human pathogen that causes typhoid fever. It is a major cause of morbidity and mortality in developing countries and is responsible for several outbreaks in developed countries. Studying certain parameters of the pathogen, such as the incubation period, provides a better understanding of its pathophysiology and its characteristics within a population. Outbreak investigations and human experimental studies provide an avenue to study these relevant parameters.

**Methods:**

In this study, the authors have undertaken a systematic review of outbreak investigation reports and experimental studies, extracted reported data, tested for heterogeneity, identified subgroups of studies with limited evidence of heterogeneity between them and identified factors that may contribute to the distribution of incubation period.

Following identification of relevant studies, we extracted both raw and summary incubation data. We tested for heterogeneity by deriving the value of I^2^ and conducting a KS-test to compare the distribution between studies. We performed a linear regression analysis to identify the factors associated with incubation period and using the resulting *p*-values from the KS-test, we conducted a hierarchical cluster analysis to classify studies with limited evidence of heterogeneity into subgroups.

**Results:**

We identified thirteen studies to be included in the review and extracted raw incubation period data from eleven. The value of I^2^ was 84% and the proportion of KS test *p*-values that were less than 0.05 was 63.6% indicating high heterogeneity not due to chance. We identified vaccine history and attack rates as factors that may be associated with incubation period, although these were not significant in the multivariable analysis (*p*-value: 0.1). From the hierarchical clustering analysis, we classified the studies into five subgroups. The mean incubation period of the subgroups ranged from 9.7 days to 21.2 days. Outbreaks reporting cases with previous vaccination history were clustered in a single subgroup and reported the longest incubation period.

**Conclusions:**

We identified attack rate and previous vaccination as possible associating factors, however further work involving analyses of individual patient data and developing mathematical models is needed to confirm these as well as examine additional factors that have not been included in our study.

**Electronic supplementary material:**

The online version of this article (10.1186/s12879-018-3391-3) contains supplementary material, which is available to authorized users.

## Background

Typhoid fever is a systemic infection caused by the bacterium *Salmonella enterica* subsp. enterica serotype Typhi (*S*. Typhi). It has been a major human pathogen for thousands of years, and while incidence has greatly reduced in developed countries, it remains a major cause of morbidity and mortality in developing countries [1]. Laboratory diagnosis of typhoid fever involves culture and identification through biochemical and serological means. Widal agglutination test was a common method until its diagnostic capabilities were questioned [2].

The mode of transmission is faecal-oral via ingestion of contaminated food and water or contact with an infected carrier [3]. Although it is a gastrointestinal infection, diarrhoea and vomiting are atypical symptoms of typhoid fever. People suffering from typhoid fever typically complain of systemic symptoms including gradual onset of fever, general malaise, headaches and abdominal pain [4].

Prevention of typhoid fever, like most other gastrointestinal infections, includes good hand hygiene [5] and exclusion of infected food handlers. In addition, typhoid vaccine has been known to offer some protection [6].

Although typhoid fever in developed countries is mostly a sporadic disease associated with travel to endemic countries, outbreaks are still commonly reported [7, 8]. Factors such as improper cooling, inadequate heating of food, contact with contaminated raw products and infected food handlers have been implicated as contributory factors of outbreaks [9].

Investigation of outbreaks contributes to the control and reduction of the burden of disease by identifying and eliminating the source of infection. Information obtained from observational studies of outbreak investigations, particularly outbreaks with well-defined point of exposures, as well as experimental studies provide a means of studying and understanding the disease by studying certain parameters in real-life and under laboratory conditions.

Incubation period of typhoid fever, which is the time between exposure and onset of clinical symptoms, is one of the essential parameters to be studied. It is expected to be a distribution rather than a single estimate as a result of individual variation based on factors such as dose response, food matrix and host characteristics. An accurate knowledge of the incubation period distribution of typhoid fever is relevant for surveillance and implementation of public health interventions. It also contributes towards effective outbreak investigation as incorrect estimations can be misleading if they were used to determine the time of exposure [10]. Accurate knowledge of the incubation period helps in correctly classifying primary and secondary cases and exclusion of travel related cases. The incubation period also offers insights into the pathophysiology of typhoid fever and is important in conducting epidemiological and ecological studies [11].

Despite the importance of knowing the accurate incubation period distribution of typhoid fever, large organisations give a wide range with no clear indication of which durations are common and which are rare. According to the World Health Organisation (WHO), incubation periods ranging from three to sixty days have been reported [3]. The Centres for Disease Control and Prevention (CDC) reported the incubation period as three to thirty days [12].

In this study, we have systematically reviewed the literature for outbreaks of well-defined point source exposures and human experimental studies of typhoid fever with the aim of estimating the distribution of the incubation period and identify factors that may explain any variation observed. We extracted summary estimates and individual incubation period data reported, tested for the presence of heterogeneity, identified factors that may contribute to heterogeneity and defined subgroups of studies than can be combined for analysis.

## Methods

### Research questions

The questions we aimed to answer in this review were:

1. What is the distribution of incubation period of *Salmonella* Typhi in humans?
2. What factors influence the incubation period?

### Modified PICO elements

Population studies /Participants - Laboratory confirmed cases of *Salmonella* Typhi in a point source exposure outbreak or continuous source outbreaks where date of exposure and onset is known for each case or experimental study.

Probable cases of typhoidal salmonellosis based on clinical presentation and case definitions in the context of a point source exposure outbreak.

Infectious agent - *Salmonella enterica* subsp. enterica serotype Typhi.

Comparator - Host factors and any other factors such as vaccine history, ingested dose, food vehicle, case definitions.

Outcome - Time from exposure to onset of clinical illness as described or defined by the authors including fever, abdominal pain, vomiting etc.

### Literature search

The authors carried out a systematic literature search of peer reviewed publications on PubMed to identify observational studies and experimental studies reporting incubation period. Combining all the terms of interest, we formed the compound search string: *Salmonella* Typhi AND Humans AND (Outbreak* OR Experiment*). The reference list of review papers was also screened to identify other relevant studies that may have been missed in the original search. The search was carried out between 29 May to 24 June 2017.

### Selection process

Each article went through a rigorous selection process, and relevant articles were assessed for the quality of reported data. The selection and assessment process were done in the following phases:

1. Screening of titles and abstracts for articles reporting typhoidal salmonellosis
2. Screening of full texts for reporting of incubation period
3. Review of full texts to assess exposure times and quality of reported data according to defined quality assessment criteria.

The quality assessment criteria used in this review has previously been explained in Awofisayo-Okuyelu et al. [13]. The process involved two reviewers independently assessing each study and comparing results. Where there was a difference in opinions, discussions were held until a consensus was reached.

### Data extraction process

Relevant data was extracted using a pre-developed pro forma (Table 1). The type of data extracted from all studies included general information on the article, characteristics of the observational study or experimental study, details of the organism, attack rate, setting of exposure, details of case definition and summary measures of the incubation period such as mean, median, mode and range where available.

**Table 1:** Details of data extracted

Section	Information to be collected
General information	- Year of publication - Title of article - Authors - Type of publication (journals, conference abstract, grey literature, etc.) - PubMed ID (where applicable)
Study characteristics	- Year of study - Study design (cohort, case-control, experimental, case series) - Country of study - Age distribution - Comments on method or quality of study
Pathogen characteristics	- Infectious agent - Species - Subtype
Outcome data/ results	- Case definition - Reported incubation period (individual data, mean, median mode and range) - Derived or calculated summary estimates incubation period (raw data extracted) - Source of calculated data (epidemic curve or author description)
Other outcome data	- Incubation period to particular symptoms
Factors that could affect incubation period	- No of exposed cases - No of people affected - Setting - Mode of transmission - Food vehicle (for foodborne infections only) - Patient characteristics (e.g. age, previous infection or treatment, underlying illness)
Any other relevant information	- Any other relevant information

Some studies further reported individual incubation data either as an epidemic curve or descriptive table. Where an epidemic curve was provided, we used a free online data extraction tool called WebPlotDigitizer version 3.10 [14] to extract the individual incubation data. If data was provided as a descriptive summary table, individual incubation data was equally extracted.

The unit of measurement reported and extracted was in days.

### Descriptive analyses

We calculated frequencies and percentages, summarising the characteristics of all studies. Characteristics summarised included: study design (observational or experimental), study type for observational studies (case-control, cohort or descriptive), year of study, country of study, age distribution of cases, mode of transmission and setting of exposure.

Using the extracted individual incubation data, we re-created the epidemic curves of the studies plotting each graph on a standard x-axis indicating incubation period ranging from zero to forty-five days and individual y-axis indicating number of cases in the outbreak or experimental study.

### Statistical analyses

Using the individual incubation data, we tested for the presence of heterogeneity and defined the pattern of heterogeneity. Using both the individual and summary incubation data, we identified factors that may explain heterogeneity. Analyses were carried out using the statistical software R version 3.2.3 (2015-12-10) – “Wooden Christmas Tree” [15]. Further details on the statistical analyses is described in Awofisayo-Okuyelu et al. [13].

### Testing for heterogeneity

We tested for heterogeneity across the studies in two ways. First, we calculated the value of I^2^ by deriving the Q statistic and inputting it into the formula: *I*^2^ = 100 % *X* (*Q* − *df*)/*Q*; where Q is the Cochran’s heterogeneity statistic and *df* is the corresponding degree of freedom. A *p*-value of less than 0.05 from the Q statistic test provided evidence of heterogeneity between the studies. Furthermore, the value of I^2^ was interpreted according to the Cochran suggested threshold [16] to determine the magnitude of heterogeneity.

The second way we tested for heterogeneity was by performing a two-sample Kolmogorov-Smirnov test (KS test) which compares the cumulative distributions between the studies. We applied a bootstrapped version of the function, repeating the sampling 100,000 times in order to derive *p*-values that will provide improved coverage due to potential ties in the data comparisons.

The output of the KS test was the D-statistic and the corresponding *p*-value. A high d-statistic value and a low *p*-value indicated the presence of heterogeneity. We further compared the *p*-values to confirm if any observed heterogeneity was due to chance. We calculated the proportion of *p*-values below 0.05 and the probability of obtaining the observed proportion. Statistical evidence of heterogeneity in the reported incubation period was available if the probability was less than 0.01.

### Identifying factors that explain heterogeneity

We performed a linear regression analysis using the summary incubation data available from all studies. We fitted a generalised linear model with gamma as the family function to account for skewness of the data and the link function used was ‘identity’. The effect of the explanatory variables on the mean incubation period was examined using a univariate model, and where there was a significant association (*p*-value 0.05), the associated variables were included in a multivariable model to test for confounding.

### Identifying subgroups of studies/describing pattern of heterogeneity

Once heterogeneity was confirmed, the next step was to identify subgroups of studies with limited evidence of heterogeneity. We used hierarchical clustering analysis to statistically identify subgroups of studies. Studies that reported only summary data (studies 7 and 11) or aggregated raw data (studies 1 and 3) were excluded from the cluster analysis.

The *p*-value of the KS test was converted to a dissimilarity matrix and used to create a hierarchical cluster to show a graphical representation of the dissimilarities between the studies. The cluster analysis algorithm used was the complete linkage method. The output was a dendrogram showing a compact visualisation of the dissimilarity matrix.

In order to identify clusters within the dendrogram, we had to determine a cut-off point at which the studies were significantly allocated to subgroups. Considering that the KS test was a pairwise test, thereby increasing the likelihood of a type 1 error (observing one significant result due to chance), we applied a pragmatic adjustment to the significance level (0.05) by dividing it by the number of studies included in the KS test. We then subtracted the corrected *p*-value from one to derive a cut-off point from which studies with limited evidence of heterogeneity can be defined within subgroups.

### Subgroup analysis

Individual incubation period data was pooled to generate a dataset for each subgroup. Summary statistics and outcome measures were derived including:

- Number of studies included in a subgroup
- Total number of cases (sum of cases in all studies included in a subgroup)
- Mean and median incubation period of cases within a subgroup
- Variance, skew and kurtosis of incubation period of cases within a subgroup
- Mean attack rate of studies within a subgroup
- Reported vaccination history of studies within a subgroup

A forest plot showing the distribution of the mean incubation period and the corresponding 95% confidence interval was created. The reported mean incubation period was used to allocate studies without individual patient data to appropriate subgroups for illustration.

### Risk of bias

We analysed our data for the presence of small-study effect using a funnel plot to visualise the relationship between sample size and incubation period.

## Results

### Literature search and selection process

**Fig. 1:**
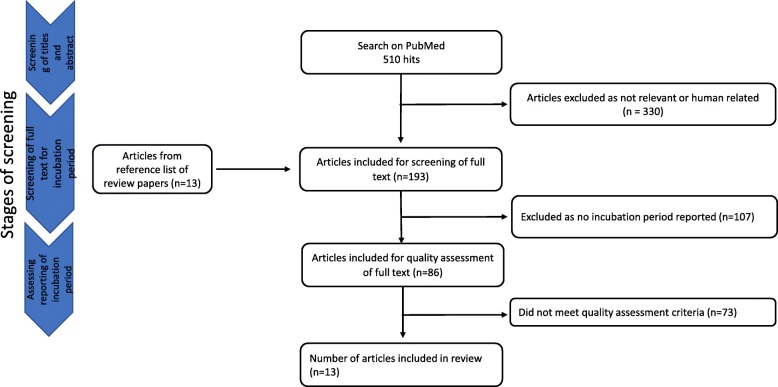
Flowchart of study selection process

A total of 510 articles were retrieved following the search in PubMed. Titles and abstracts were screened to identify relevant articles. Articles were excluded if there were non-human related studies, or non-typhi studies. Studies that were not reported in English were also excluded. This resulted in 180 articles available for full text screening of incubation period data after excluding 330 irrelevant articles. Searching through the reference list of other review papers, we identified thirteen articles that were not included in the original search, bringing the number of articles available for full text screening to 193. Excluding articles that did not report incubation period resulted in 86 articles that went through the quality assessment process. Seventy-three articles did not meet the quality assessment criteria (Additional file 1) and were further excluded (Fig. 1). The resulting number of articles available for inclusion in the study was thirteen (Additional file 2), all of which reported individual incubation data with the exception of studies 7 and 11.

### Descriptive analysis of studies

All studies included in our review reported incubation period of cases associated with *S*. Typhi, however, very few studies reported the microbiological characteristics (phage types) of the infecting organism.

Studies included in our review were published between 1914 and 2016. Sixty one percent (8/13) of the outbreaks or experiments took place before year 2000 with the majority (5/13; 38.5%) occurring the North America (Table 2). Two studies were experimental and the remainder were epidemiological studies including descriptive studies (4/13; 30.8%), case control studies (4/13; 30.8%) and retrospective cohort studies (3/13; 23.1%). The eleven outbreaks included resulted in a total of 635 cases.

**Table 2:** Characteristics of included studies

	N	%
Total number of studies	13	
Year of study		
Before year 2000	8	61.5
2000 and later	4	30.8
unknown	1	7.7
Region of study		
Europe	3	23.1
North America	5	38.5
Africa	2	15.4
Asia	2	15.4
Unknown	1	7.7
Study design		
Descriptive	4	30.8
Case control	4	30.8
Retrospective cohort	3	23.1
Experimental study	2	15.4
Setting of exposure		
Catered meal	5	38.5
Outdoor activity	2	15.4
Picnic	2	15.4
Experimental study	2	15.4
Community	1	7.7
Restaurants	1	7.7
Food vehicle category		
Red meat	2	15.4
Dairy and dairy products	2	15.4
Salad	3	23.1
Non-foodborne	1	7.7
Other	3	23.1
Unknown	2	15.4

The settings of exposure varied and the most common reported setting was events with catered meals (5/13; 38.5%). Outdoor activities and picnic events were reported in two outbreaks each (2/13; 15.4%). With the exception of one outbreak that was caused by exposure to surface water at a recreational park, all infections were foodborne and the most frequently reported food vehicles were salad and vegetables (3/13; 23.1%), red meat and dairy both accounting for two outbreaks each (2/13; 15.4%). The food vehicle for two outbreaks was unknown. (Table 2).

The funnel plot did not indicate any effect of study size on the reported incubation period as it was symmetric and the data points were evenly distributed around the x-axis (Additional file 3).

**Fig. 2:**
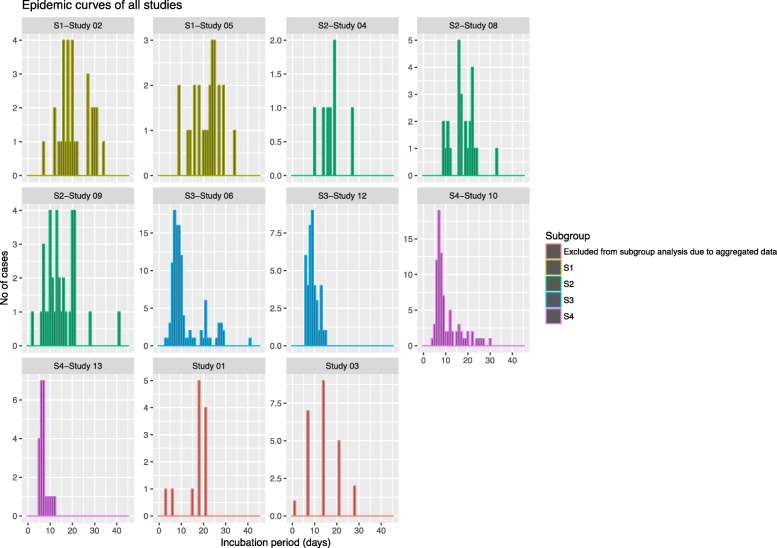
Collated epidemic curves of eleven studies re-created from raw data and arranged according to subgroup

Reviewing the re-created epidemic curves from the eleven studies that provided individual incubation data, we observed a range of distributions in the plots (Fig. 2). The mean incubation period ranged from 7 days to 21.4 days. The minimum incubation period reported was 2 days and the maximum was 41 days (Table 3).

**Table 3:** Additional characteristics of included studies

Study number	Mean incubation period	No. affected (attack rate)	No hospitalised (deaths)	Age Distribution	Source	Setting of exposure	Food vehicle	Ingested dose	Vaccine history	Case definitions as described by the studies
Study 1	16.5	12 (Low)	0 (0)	children	Surface water	Recreational park	NR	Not reported	Not reported	All clinical symptoms with or without microbiological confirmation
Study 2	20.9	31 (31.63)	16 (0)	Mixed	Food handler	catered meal	Sandwiches	Not reported	17 cases reported previous vaccination at anytime	All clinical symptoms
Study 3	14	24 (40)	7 (0)	Mixed	Food handler	Picnic	Potato Salad	Not reported	None	All clinical symptoms with or without microbiological confirmation
Study 4	17.4	7 (NR)	7 (0)	Mixed	Food handler	Restaurant	NR	Not reported	Not reported	All clinical symptoms
Study 5	21.4	24 (25.53)	24 (0)	Adults	Food handler	Outdoor activity	Cucumber salad	Not reported	All cases	Specific to fever with or without microbiological confirmation
Study 6	11.4	235 (34.97)	NR	Adults	Experimental	Experimental	Milk	10^5^	None	Specific to fever
Study 7	20.6	69 (65.09)	69 (0)	Mixed	Food handler	catered meal	NR	Not reported	Not reported	All clinical symptoms with or without microbiological confirmation
Study 8	17.7	26 (7.42)	26 (0)	NR	Unknown	catered meal	Pork meat	Not reported	Not reported	Specific to fever with or without microbiological confirmation
Study 9	14.7	35 (14)	35 (6)	Mixed	Food handler	catered meal	Vegetable	Not reported	Not reported	All clinical symptoms with or without microbiological confirmation
Study 10	10.5	84 (56)	77 (3)	Mixed	Food handler	catered meal	Spanish spaghetti	Not reported	Not reported	All clinical symptoms with or without microbiological confirmation
Study 11	9.7 & 7.6	24 (60)	0 (0)	Adults	Experimental	Experimental	Sodium bicarbonate	10^3^ & 10^4^	None	Specific to fever with or without microbiological confirmation
Study 12	9.1	41 (73.21)	NR	Mixed	Unknown	community	Corned beef	Not reported	Not reported	All clinical symptoms
Study 13	7	23 (79.31)	23 (3)	NR	Food handler	Picnic	Ice cream	Not reported	Not reported	Specific to fever and excluding diarrhea and vomiting

*NR* Not reported

Identification of cases was dependent on the case definition given by the authors of each study. In eight studies, microbiological confirmation was used to define cases, in two studies (studies 6 and 13), a very specific case definition including epidemiological link was used and in three studies, the case definition was less specific but also including epidemiological link (Table 3).

Of the twelve studies where the attack rates could be calculated, five had an attack rate higher than 50% and the highest attack rate was 79.31% in an outbreak involving ingestion of contaminated ice cream [17]. This outbreak also recorded the second highest case fatality rate.

Five studies reported vaccination history of cases, and of these, two reported previous vaccination of some or all of their cases. These two studies reported the longest mean incubation period of 21.4 and 20.9 days. The attack rates in these populations were below 50%, however, these were not the lowest attack rates reported.

### Test for heterogeneity

The *p*-value of the derived Q statistic was < 0.0001 and the calculated value of I^2^ was 84% indicating high heterogeneity in the reported incubation period across the studies. The proportion of KS test *p*-values that were less than 0.05 was 63.6% (42/66) and the probability of obtaining the observed proportion was < 0.0001 further confirming the high heterogeneity across the studies which is not due to chance.

### Factors that may explain heterogeneity

Results of the univariate analysis showed that previous vaccination history, attack rate and year of study had a significant association with the mean incubation period. The mean incubation period reduced by 1.4 days with every 10% increase in attack rate (*p*-value 0.05), and outbreaks containing cases that had been vaccinated reported a longer incubation period of 9.8 days compared to outbreaks without vaccinated cases (Table 4). Outbreaks that occurred after 1950 reported a longer incubation period of 6.8 days compared to outbreaks that occurred before 1950. When these variables were included in the multivariable analysis, there was no significant association between vaccination, year of study and mean incubation. The effect observed for year of study was reversed such that outbreaks that occurred after 1950 now reported a shorter incubation period of 3.9 days.

**Table 4:** Linear regression analysis to identify factors associated with the distribution of incubation period

Variables	Univariate analysis		Multivariable analysis	
	Difference in mean	*p*-value	Difference in mean	*p*-value
Age distribution				
Adult	Reference			
Children	2.7	0.6		
Mixed ages	1.5	0.6		
Year of study				
Pre 1950	Reference			
Post 1950	6.8	0.01	−3.9	0.1
Attack rate	−0.14	0.05	−0.26	0.07
Setting				
Restaurant	Reference			
Catered meal	−0.5	0.9		
Community	−8.3	0.1		
Experimental	−7.4	0.2		
Outdoor activity	1.5	0.8		
Picnic	−6.9	0.2		
Food category				
Other	Reference			
Dairy	−4.3	0.3		
Non-food	3.1	0.6		
Red meat	0.1	0.9		
Salad & vegetable	3.3	0.5		
Vaccine history				
None	Reference			
Yes	10.3	0.03	4.2	0.1
Specific case definition				
No	Reference			
Yes	−2.2	0.4		

### Identifying subgroups of studies

**Fig. 3:**
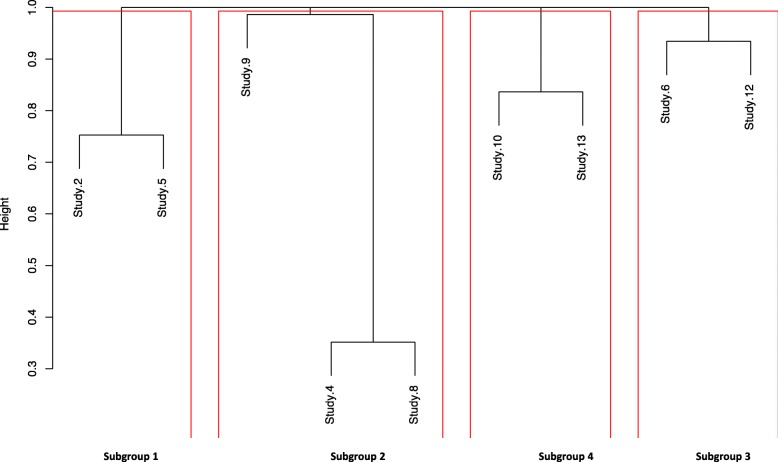
Dendrogram showing compact visualization of dissimilarity matrix indicating pattern of heterogeneity and highlighting subgroups of the studies that can be combined for further analysis

Based on the clustering analysis, studies were paired according to the evidence of dissimilarities between them. Studies found to have the least evidence of dissimilarities were paired and then connected by branches to another pair of studies or a single study with minimal dissimilarity to them. These pairing and connections of studies create a dendrogram of the dissimilarity matrix (Fig. 3).

The pragmatic adjustments made to the significance level resulted in a corrected *p*-value of 0.004 and a corresponding cut-off point of 0.994. At this cut-off point, after taking into account multiple testing, four subgroups were identified. These included: one subgroup of three studies and three subgroups of two studies each (Fig. 3).

### Summary of subgroup analysis

**Fig. 4:**
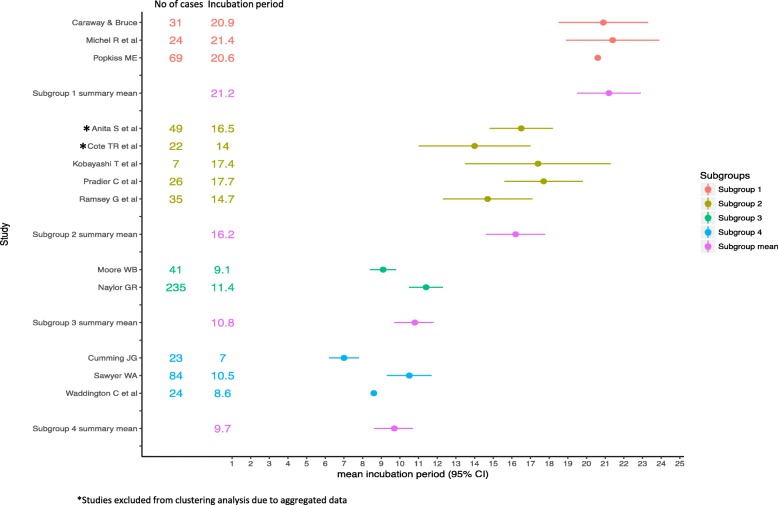
Forest plot showing mean incubation period of included studies and subgroup summary mean and 95% confidence intervals

The mean incubation period was different between the subgroups (Fig. 4) and significantly decreased from subgroup 1 to subgroup 3. Subgroup 4 had the shortest mean incubation period of 9.7 days (95% CI 8.6–10.7) and subgroup 1 had the longest mean of 21.2 days (95% CI 19.5–22.9) (Table 5). We also observed some differences in the variance, skew and kurtosis between subgroups. Subgroups 3 and 4 had the highest mean attack rate and also had the shortest incubation periods. Cases in subgroup 1 reported previous vaccination and also reported the longest incubation period.

**Table 5:** Summary statistics of outbreaks and incubation period within subgroups

	Count	Sum of cases	Median	Mean (95% CI)	Variance	Skew	Kurtosis	Attack rate	Vaccine
Subgroup 1	2	55	20	21.2 (19.5–22.9)	42.2	−0.002	−0.6	28.7	All or most cases
Subgroup 2	3	68	16	16.2 (14.6–17.8)	42.7	0.9	2.3	NR	Unknown
Subgroup 3	2	276	9	10.8 (9.7–11.8)	39.7	2.1	4.4	54.1	None
Subgroup 4	2	107	8	9.7 (8.6–10.7)	29.3	1.8	2.9	67.6	Unknown

*NR* Not reported

## Discussion

We have undertaken a systematic review to describe the distribution of incubation period of *Salmonella* Typhi. We tested for the presence of heterogeneity, attempted to identify factors that could influence the incubation period and classified the studies into subgroups with limited evidence of heterogeneity. Due to the high heterogeneity amongst the studies, estimating a single distribution of incubation period was impossible. However, we defined subgroups of similar studies and found their mean incubation period to range from 9.7 days to 21.2 days. These values are within the range quoted by international organisations reporting 3 to 60 days [3], 3 to 30 days [12] and also scientific literatures reporting 10 to 20 days [18]. Some literature report shorter incubation periods than we observed, like Raffatellu et al. reporting a median of 5 to 9 days [19] or 8 to 14 days [18]. We also identified attack rate and vaccination as possible factors that could affect the incubation period distribution.

The relationship between attack rate and incubation period is inversely proportional, such that higher attack rates result in shorter incubation periods [20, 21]. This has been reported in numerous outbreaks [22–24] and also observed in our review. Attack rates are associated with factors such as virulence of the organism, host characteristics and infecting dose [22]. When either of these factors are present in a way that increases the attack rate: high virulence, susceptible host and large infecting dose, the incubation period is shortened as the onset of illness is quicker.

Vaccination is one of the preventive measures advised by public health professionals [25, 26] to control or mitigate the burden of disease brought on by *S*. Typhi [27]. It is recommended to people living in or visiting endemic areas and to control outbreaks [3]. Our study did not aim to review the efficacy of typhoid vaccine in preventing outbreaks, however we identified a couple of studies with outbreaks reported in vaccinated population [28, 29] and similar studies have also been reported [30]. As observed from our review, we may say that in populations where vaccination does not prevent illness, it may delay the onset of symptoms thereby prolonging the incubation period. This could have an adverse effect in outbreaks with continuous source as more people will be exposed and possibly infected before the outbreak is discovered.

The subgroup analysis indicated that outbreaks and experimental studies that took place before the year 1950 belonged to subgroups reporting shorter incubation period (subgroups 3 and 4). In the univariate analysis, post-1950 studies had a significantly longer incubation period, however, in the multivariate analysis, this effect on the incubation period was reversed as we observed a shorter incubation period in post-1950 studies. The change in the direction of effect is indicative of the presence of a confounding factor in the univariate analysis.

A lot of reported outbreaks caused by *S*. Typhi either had a continuous source of exposure such as municipal water supply [31–34], or were caused by widely distributed food produce [35, 36]. This means that point source outbreaks, where the time of exposure is definite and incubation period could be accurately calculated, were few. This was a similar finding in the review conducted by Naylor [24]. The author stated the criteria of an outbreak suitable for incubation period analysis and reported that very few reports fulfilled these criteria. However, in their review, studies where the date of exposure was not confirmed were included and date of infection was inferred from date of purchase, date of sale for a batch of food item, date of water contamination and so on. Continuous source outbreaks can be useful if the investigators identify and report the exact dates of exposure and onset but this was not the case in the outbreaks reviewed by Naylor. Hence, some studies identified in Naylor’s review were not included in our study. In our study, we identified only 13 studies with point source exposures or where the time of exposure was known. Some studies however reported point source exposures, although the exposures could have occurred over a few days [37, 38].

Dose response is known to be associated with incubation period [20, 39], and although this was not particularly studied in our review, there were studies were this relationship was observed. In an outbreak associated with ice cream [17], a dose response relationship was observed as those who had a mixture of ice creams had longer incubation periods while those who had only the contaminated ice cream had comparatively shorter incubation periods. Another dose response relationship was observed in the experimental study conducted by Waddington et al. [21]. Two cohorts of volunteers were infected with different doses of inoculum, 10^3^ and 10^4^. The cohort receiving the lower dose had a longer incubation period compared to those receiving the higher dose.

The case definition used in identifying cases or in including cases as part of an outbreak is very subjective and based on what the authors identify as a case. This is quite essential because it can either prolong or shorten the incubation period of a case based on what is considered as the onset of symptoms. Although there are guidelines on what to consider when defining a case [40], case definitions depend on the authors’ perception. The complex syndrome of typhoid fever [7] makes the process of defining cases arbitrary. In our review, some of the case definitions were broad and syndromic, such as ‘clinical illness’, while others are very specific in terms of fever at a particular temperature [21, 29, 41] or dismissing symptoms such as diarrhoea and vomiting [17]. Neither the broad nor specific definitions are incorrect, however, the varied case definitions may have contributed to the observed heterogeneity.

A limitation we encountered in this review was the difference in the reported incubation period data extracted from the included studies. Although the raw incubation times were reported in days, some studies reported an aggregated date of symptom onset in three or seven-day intervals [42, 43]. Onset times were either rounded up or rounded down to fall into the reported interval. Rounding down the onset time from three days to one will spuriously shorten the incubation period just as rounding up the onset time from one day to seven will spuriously prolong the incubation period. We therefore excluded these studies from the hierarchical cluster analysis and their reported mean incubation periods should be interpreted with caution.

## Conclusions

Our study showed that the reported incubation period varied more than could be explained by chance and identified attack rate and previous vaccination as associated factors, although not significantly. This may be due to the limited information available from the small number of studies we reviewed. Analysing individual patient data will provide an opportunity to assess additional patient characteristics such as underlying medical conditions, drug interactions and full vaccination history. In addition, using mathematical models to describe the process of infection will help identify parameters intrinsic to the infection pathway that could influence the distribution of incubation period such as gastric transit times, phagocyte characteristics and cellular bacteria growth.

In order to maximise the use of outbreak reports in studying parameters such as incubation period, authors should endeavour to precisely report exposure times and onset times. Where the outbreak is non-point source, identifying and reporting the exposure time of each case where possible will be useful. Case definitions should be developed in such a way as to ensure that cases are included based on similar thresholds which can also be comparable with other outbreak reports.

## Additional files

Additional file 1: List of studies excluded from the review. Table listing the studies excluded from the review. (DOCX 24 kb)

Additional file 2: List of included studies and data. Spreadsheet containing a list of studies included in the review and the data extracted from the studies. (XLSX 14 kb)

Additional file 3: Funnel plot. Graph of funnel plot showing the effect of study size on the incubation period. (PDF 5 kb)

Additional file 4: PRISMA 2009 checklist. Table of PRISMA checklist. (DOC 73 kb)

## Abbreviations

KS test: Kolmogorov-Smirnov test
NR: Not reported
