# Supplementary material for: Incubation period of typhoidal salmonellosis: a systematic review and meta-analysis of outbreaks and experimental studies occurring over the last century
Source: BMC Infect Dis. 2018 Sep 27;18:483. doi: 10.1186/s12879-018-3391-3 (PMC6161394; doi:10.1186/s12879-018-3391-3)
Supplement: Supplementary file 1 — List of studies excluded from the review. Table listing the studies excluded from the review. (DOCX 24 kb) [file 12879_2018_3391_MOESM1_ESM.docx]

List of studies excluded from the review

| **Article** | **Reason for exclusion** |
| --- | --- |
| Agarwal V, Jalgaonkar PD, Pathak AA, Saoji AM. An outbreak of multidrug resistant typhoid fever in Nagpur. J Assoc Physicians India. 1992 Jun;40(6):416. | No incubation period reported |
| Al-Sanouri TM, Paglietti B, Haddadin A, Murgia M, Bacciu D, Youssef M, et al. Emergence of plasmid-mediated multidrug resistance in epidemic and non-epidemic strains of *Salmonella enterica* serotype Typhi from Jordan. J Infect Dev Ctries. 2008 Aug 30;2(4):295–301. | Study on microbiology of *S*. Typhi with no report on incubation period |
| al-Zubaidy AA, el Bushra HE, Mawlawi MY. An outbreak of typhoid fever among children who attended a potluck dinner at Al-Mudhnab, Saudi Arabia. East Afr Med J. 1995 Jun;72(6):373–5. | Exposure could have occurred over two days or one of two days |
| Anderson ES, Hobbs BC. Studies of the strain of *Salmonella* Typhi responsible for the Aberdeen typhoid outbreak. Isr J Med Sci. 1973 Feb;9(2):162–74. | Study on microbiology of *S*. Typhi with no report on incubation period |
| Arora RK, Gupta A, Joshi NM, Kataria VK, Lall P, Anand AC. Multidrug resistant typhoid fever: study of an outbreak in Calcutta. Indian Pediatr. 1992 Jan;29(1):61–6. | Study on microbiology of *S*. Typhi with no report on incubation period |
| Arya SC, Agarwal N. Re: outbreak of typhoid fever in vaccinated members of the French Armed Forces in the Ivory Coast. Eur J Epidemiol. 2006;21(5):407. | Microbiological focus of a study included in the review |
| Ash I, Mckendrick GD, Robertson MH, Hughes HL. Outbreak of typhoid fever connected with corned beef. Br Med J. 1964 Jun 6;1(5396):1474–8. | Non-point source outbreak. Corned beef purchased on a particular day, but unsure of date of consumption. |
| Aye TT, Siriarayapon P. Typhoid fever outbreak in Madaya Township, Mandalay Division, Myanmar, September 2000. J Med Assoc Thai. 2004 Apr;87(4):395–9. | Non-point source outbreak. |
| Ayliffe GA, Geddes AM, Pearson JE, Williams TC. Spread of *Salmonella* Typhi in a maternity hospital. J Hyg (Lond). 1979 Jun;82(3):353–9. | Source of exposure and mode of transmission unknown. No incubation period reported. |
| Aypak A, Celik AK, Aypak C, Cikman O. Multidrug resistant typhoid fever outbreak in Ercek Village-Van, Eastern Anatolia, Turkey: clinical profile, sensitivity patterns and response to antimicrobials. Trop Doct. 2010 Jul;40(3):160–2. | No incubation period reported |
| Ayyagari A, Pal N. Outbreak of typhoid fever due to multi-resistant *Salmonella* Typhi in northern India--a preliminary report. Trans R Soc Trop Med Hyg. 1991 Apr;85(2):302. | No incubation period reported |
| Bahrmand AR, Velayati AA. Antimicrobial resistance pattern and plasmid profile of *Salmonella* Typhi isolated from an outbreak in Tehran province. Scand J Infect Dis. 1997;29(3):265–9. | Study on microbiology of *S*. Typhi with no report on incubation period |
| Bayram Y, Güdücüoğlu H, Otlu B, Aypak C, Gürsoy NC, Uluç H, et al. Epidemiological characteristics and molecular typing of *Salmonella* *enterica* serovar Typhi during a waterborne outbreak in Eastern Anatolia. Ann Trop Med Parasitol. 2011 | Non-point source outbreak. |
| Bhunia R, Hutin Y, Ramakrishnan R, Pal N, Sen T, Murhekar M. A typhoid fever outbreak in a slum of South Dumdum municipality, West Bengal, India, 2007: evidence for foodborne and waterborne transmission. BMC Public Health. 2009 Apr 27;9:115. | Non-point source outbreak and possible secondary transmission. |
| Bissett ML, Powers C, Wood RM. Immunofluorescent identification of *Salmonella* Typhi during a typhoid outbreak. Appl Microbiol. 1969 Apr;17(4):507–11. | Study on microbiology of *S*. Typhi with no report on incubation period |
| Blum LS, Dentz H, Chingoli F, Chilima B, Warne T, Lee C, et al. Formative investigation of acceptability of typhoid vaccine during a typhoid fever outbreak in Neno District, Malawi. Am J Trop Med Hyg. 2014 Oct;91(4):729–37. | Qualitative study on vaccine acceptability. No incubation period reported. |
| Bradarić N, Punda-Polić V, Milas I, Ivić I, Grgić D, Radosević N, et al. Two outbreaks of typhoid fever related to the war in Bosnia and Herzegovina. Eur J Epidemiol. 1996 Aug;12(4):409–12. | Unable to determine time of exposure |
| Bradley WH, Evans LW, Taylor I. A hospital outbreak of typhoid fever. J Hyg (Lond). 1951 Sep;49(2–3):324–34. | Exposure seemed to have occurred over 3 days |
| Centers for Disease Control and Prevention (CDC). Notes from the field: *Salmonella* Typhi infections associated with contaminated water--Zimbabwe, October 2011-May 2012. MMWR Morb Mortal Wkly Rep. 2012 Jun 15;61(23):435. | Non-point source outbreak. |
| Coovadia YM, Gathiram V, Bhamjee A, Garratt RM, Mlisana K, Pillay N, et al. An outbreak of multi-resistant *Salmonella* Typhi in South Africa. Q J Med. 1992 Feb;82(298):91–100. | Study on microbiology f *S*. Typhi with no report on incubation period |
| Coupee WRM, Newell KW, Payne DJH. An Outbreak of Typhoid Fever associated with Canned Ox-Tongue. Lancet. 1956;1057–9. | Contaminated product bought over two weeks, even though author claims exposure occurred on one day |
| Egoz N, Shihab S, Leitner L, Lucian M. An outbreak of typhoid fever due to contamination of the municipal water supply in northern Israel. Isr J Med Sci. 1988 Nov;24(11):640–3. | Non-point source outbreak. Community supplied water |
| Engleberg NC, Barrett TJ, Fisher H, Porter B, Hurtado E, Hughes JM. Identification of a carrier by using Vi enzyme-linked immunosorbent assay serology in an outbreak of typhoid fever on an Indian reservation. J Clin Microbiol. 1983 Dec;18(6):1320–2. | Exposure time unsure. No incubation period reported |
| Farooqui A, Khan A, Kazmi SU. Investigation of a community outbreak of typhoid fever associated with drinking water. BMC Public Health. 2009 Dec 20;9:476. | Non-point source outbreak. |
| Gallay A, Vaillant V, Bouvet P, Grimont P, Desenclos JC. How many foodborne outbreaks of *Salmonella* infection occurred in France in 1995? Application of the capture-recapture method to three surveillance systems. Am J Epidemiol. 2000 Jul 15;152(2):171–7. | Review of a surveillance system |
| Glynn JR, Bradley DJ. The relationship between infecting dose and severity of disease in reported outbreaks of *Salmonella* infections. Epidemiol Infect. 1992 Dec;109(3):371–88. | Dose response study. No incubation period reported |
| Goh KT, Teo SH, Tay L, Monteiro EH. Epidemiology and control of an outbreak of typhoid in a psychiatric institution. Epidemiol Infect. 1992 Apr;108(2):221–9. | Non-point source outbreak. |
| Gonzalez-Cortes A, Bessudo D, Sanchez-Leyva R, Fragoso R, Hinojosa M, Becerril P. Water-borne transmission of chloramphenicol-resistant *Salmonella* Typhi in Mexico. Lancet. 1973 Sep 15;2(7829):605–7. | Non-point source outbreak. |
| Gupta V, Kaur U, Singh G, Prakash C, Sharma M, Aggarwal KC. An outbreak of typhoid fever in Chandigarh, North India. Trop Geogr Med. 1986 Mar;38(1):51–4. | Non-point source outbreak. |
| Halder KK, Dalal BS, Ghose E, Sanyal S. Chloramphenicol resistant *Salmonella* Typhi: the cause of recent out-break of enteric fever in Calcutta. Indian J Pathol Microbiol. 1992 Jan;35(1):11–7. | No incubation period reported |
| Hancock-Allen J, Cronquist AB, Peden J, Adamson D, Corral N, Brown K. Notes from the field: typhoid fever outbreak associated with an asymptomatic carrier at a restaurant - Weld County, Colorado, 2015. MMWR Morb Mortal Wkly Rep. 2016 Jun 17;65(23):606–7. | Non-point source outbreak. |
| Hardy G, Padfield CJ, Chadwick P, Partington MW. Typhoid outbreak in Kingston, Ont: experience with high-dose oral ampicillin. Can Med Assoc J. 1977 Apr 9;116(7):761–4, 767. | Undefined exposure time |
| Hendriksen RS, Leekitcharoenphon P, Lukjancenko O, Lukwesa-Musyani C, Tambatamba B, Mwaba J, et al. Genomic signature of multidrug-resistant *Salmonella enterica* serovar Typhi isolates related to a massive outbreak in Zambia between 2010 and 2012. J Clin Microbiol. 2015 Jan;53(1):262–72. | Study on microbiology of *S*. Typhi with no report on incubation period |
| Henriksen SD, Riddervoid HO. An outbreak of food-poisoning due to an unusual strain of *Salmonella* Typhi. Acta Pathologica Et Microbiologica Scandinavica 1952 Jun; 93:254-258. | Possible point source outbreak, however, no human confirmation and atypical of salmonella typhi. Most likely not *S*. Typhi outbreak. Author also mentioned that there are no clinical typhoid cases. |
| Hoffman TA, Ruiz CJ, Counts GW, Sachs JM, Nitzkin JL. Waterborne typhoid fever in Dade County, Florida. Clinical and therapeutic evaluation of 105 bacteremic patients. Am J Med. 1975 Oct;59(4):481–7. | Non-point source outbreak. |
| Jain S, Chitnis DS, Sham A, Rathi S, Inamdar S, Rindani GJ. Outbreak of chloramphenicol resistant typhoid fever. Indian Pediatr. 1987 Mar;24(3):193–7. | Study on microbiology of *S*. Typhi with no report on incubation period |
| Jordan EO, Irons EE. The Rockford (Ill) typhoid epidemic. J Infect Dis. 1912;11:21–43. | Unknown exposure time. Difficult to distinguish primary from secondary cases |
| Kamili MA, Ali G, Shah MY, Rashid S, Khan S, Allaqaband GQ. Multiple drug resistant typhoid fever outbreak in Kashmir Valley. Indian J Med Sci. 1993 Jun;47(6):147–51. | Non-point source outbreak. |
| Kato Y, Fukayama M, Adachi T, Imamura A, Tsunoda T, Takayama N, et al. Multidrug- resistant typhoid fever outbreak in travelers returning from Bangladesh. Emerging Infect Dis. 2007 Dec;13(12):1954–5. | Non-point source outbreak. |
| Katz DJ, Cruz MA, Trepka MJ, Suarez JA, Fiorella PD, Hammond RM. An outbreak of typhoid Fever in Florida associated with an imported frozen fruit. J Infect Dis. 2002 Jul 15;186(2):234–9. | Non-point source outbreak. |
| Keddy KH, Sooka A, Ismail H, Smith AM, Weber I, Letsoalo ME, et al. Molecular epidemiological investigation of a typhoid fever outbreak in South Africa, 2005: the relationship to a previous epidemic in 1993. Epidemiol Infect. 2011 Aug;139(8):1239–45. | Study on microbiology f *S*. Typhi with no report on incubation period |
| King CC, Chen CJ, You SL, Chuang YC, Huang HH, Tsai WC. Community-wide epidemiological investigation of a typhoid outbreak in a rural township in Taiwan, Republic of China. Int J Epidemiol. 1989 Mar;18(1):254–60. | Non-point source outbreak. |
| Kulkarni R, Bhandar M, Srinivasa S. Chloramphenicol resistant *Salmonella* Typhi outbreak in a rural part of north-east Karnataka. J Commun Dis. 1994 Dec;26(4):235–6. | No incubation period reported |
| Lewis MD, Serichantalergs O, Pitarangsi C, Chuanak N, Mason CJ, Regmi LR, et al. Typhoid fever: a massive, single-point source, multidrug-resistant outbreak in Nepal. Clin Infect Dis. 2005 Feb 15;40(4):554–61. | Single source of exposure associated with water supply, but not point source outbreak as exposure occurred over a period of months |
| Lin FY, Becke JM, Groves C, Lim BP, Israel E, Becker EF, et al. Restaurant-associated outbreak of typhoid fever in Maryland: identification of carrier facilitated by measurement of serum Vi antibodies. J Clin Microbiol. 1988 Jun;26(6):1194–7. | Time of exposure not reported |
| Loharikar A, Newton A, Rowley P, Wheeler C, Bruno T, Barillas H, et al. Typhoid fever outbreak associated with frozen mamey pulp imported from Guatemala to the western United States, 2010. Clin Infect Dis. 2012 Jul;55(1):61–6. | Non-point source outbreak. |
| Mallory A, Belden EA, Brachman PS. The current status of typhoid fever in the United States and a description of an outbreak. J Infect Dis. 1969 Jun;119(6):673–6. | Non-point source outbreak. Infection probably occurred over two days |
| Marmion DE, Naylor GRE, Stewart IO. Second attacks of typhoid fever. J Hyg (Lond). 1953 Jun;51(2):260–7. | Primary cases not well differentiated from secondary cases |
| Miner JR. The Incubation Period of Typhoid Fever. The Journal of Infectious Diseases. 1922;31(3):296–301. | Review of other outbreaks already included in the study |
| Muti M, Gombe N, Tshimanga M, Takundwa L, Bangure D, Mungofa S, et al. Typhoid outbreak investigation in Dzivaresekwa, suburb of Harare City, Zimbabwe, 2011. Pan Afr Med J. 2014;18:309. | Non-point source outbreak. |
| Neil KP, Sodha SV, Lukwago L, O-Tipo S, Mikoleit M, Simington SD, et al. A large outbreak of typhoid fever associated with a high rate of intestinal perforation in Kasese District, Uganda, 2008-2009. Clin Infect Dis. 2012 Apr;54(8):1091–9. | Non-point source outbreak. |
| Neill WA, Martin JD, Belden EA, Trotter WY. A widespread epidemic of typhoid fever traced to a common exposure. New England Journal of Medicine. 1958 Oct 2;259(14):667–72. | Date of actual exposure unknown. Date of arrival to camp was used as a proxy to calculate incubation period. |
| Olsen SJ, Bleasdale SC, Magnano AR, Landrigan C, Holland BH, Tauxe RV, et al. Outbreaks of typhoid fever in the United States, 1960-99. Epidemiol Infect. 2003 Feb;130(1):13–21. | Review study. No incubation period reported |
| Olsen SJ, Kafoa B, Win NS, Jose M, Bibb W, Luby S, et al. Restaurant-associated outbreak of *Salmonella* Typhi in Nauru: an epidemiological and cost analysis. Epidemiol Infect. 2001 Dec;127(3):405–12. | Non-point source outbreak. |
| Rajeev A. Quinolone resistant typhoid outbreak in an extended joint family. J Commun Dis. 1999 Dec;31(4):263–5. | Exposure time not stated |
| Ramanan A, Pandit N, Yeshwanth M. Unusual complications in a multidrug resistant *Salmonella* Typhi outbreak. Indian Pediatr. 1992 Jan;29(1):118–20. | Date of onset or exposure not reported |
| Rao RS, Amarnath SK, Sujatha S. An outbreak of typhoid due to multidrug resistant *Salmonella* Typhi in Pondicherry. Trans R Soc Trop Med Hyg. 1992 Apr;86(2):204–5. | Review paper with no incubation period reported |
| Rathish KC, Chandrashekar MR, Nagesha CN. An outbreak of multidrug resistant typhoid fever in Bangalore. Indian J Pediatr. 1995 Aug;62(4):445–8. | Review paper with no incubation period reported |
| Reller ME, Olsen SJ, Kressel AB, Moon TD, Kubota KA, Adcock MP, et al. Sexual transmission of typhoid fever: a multistate outbreak among men who have sex with men. Clin Infect Dis. 2003 Jul 1;37(1):141–4. | Non-GI transmission |
| Roy JS, Saikia L, Medhi M, Tassa D. Epidemiological investigation of an outbreak of typhoid fever in Jorhat town of Assam, India. Indian J Med Res. 2016 Oct;144(4):592–6. | Non-point source outbreak. Source of exposure is community water supply |
| Sejvar J, Lutterloh E, Naiene J, Likaka A, Manda R, Nygren B, et al. Neurologic manifestations associated with an outbreak of typhoid fever, Malawi--Mozambique, 2009: an epidemiologic investigation. PLoS ONE. 2012;7(12):e46099. | No incubation period reported |
| Singla N, Bansal N, Gupta V, Chander J. Outbreak of Salmonella Typhi enteric fever in sub-urban area of North India: a public health perspective. Asian Pac J Trop Med. 2013 Feb;6(2):167–8. | Non-point source outbreak and no incubation period reported |
| Stanwell-Smith RE, Ward LR. An international point source outbreak of typhoid fever: a European collaborative investigation. Bull World Health Organ. 1986;64(2):271–8. | No incubation period reported |
| Styliads S, Borczyk A. Typhoid outbreak associated with consumption of raw shellfish-- Ontario. Can Commun Dis Rep. 1994 Apr 30;20(8):63–5. | Non-point source outbreak. Food vehicle was purchased over several days |
| Swaddiwudhipong W, Kanlayanaphotporn J. A common-source water-borne outbreak of multidrug-resistant typhoid fever in a rural Thai community. J Med Assoc Thai. 2001 Nov;84(11):1513–7. | Non-point source outbreak. Source of exposure is community water supply |
| Taylor A, Santiago A, Gonzalez-Cortes A, Gangarosa EJ. Outbreak of typhoid fever in Trinidad in 1971 traced to a commercial ice cream product. Am J Epidemiol. 1974 Aug;100(2):150–7. | Non-point source outbreak. Dates ice creams were purchased or consumed is unknown. Only date of distribution is reported |
| Taylor JP, Shandera WX, Betz TG, Schraitle K, Chaffee L, Lopez L, et al. Typhoid fever in San Antonio, Texas: an outbreak traced to a continuing source. J Infect Dis. 1984 Apr;149(4):553–7. | Non-point source outbreak. |
| Torin DE, Betts SL, McClenahan JB, Phillips GM, Shikamura MT. A typhoid fever outbreak on a university campus. Arch Intern Med. 1969 Nov;124(5):606–10. | Unknown exposure, so difficult to know when cases were exposed |
| Usera MA, Aladueña A, Echeita A, Amor E, Gomez-Garcés JL, Ibañez C, et al. Investigation of an outbreak of Salmonella Typhi in a public school in Madrid. Eur J Epidemiol. 1993 May;9(3):251–4. | No specific onset dates reported |
| Weaver LT, Eccles JB, Eccles MP, Foord F. *Salmonella* Typhi infection associated with a school feeding programme. J Trop Pediatr. 1989 Dec;35(6):331–2. | Dates of onset not reported |
| Xercavins M, Llovet T, Navarro F, Morera MA, Moré J, Bella F, et al. Epidemiology of an unusually prolonged outbreak of typhoid fever in Terrassa, Spain. Clin Infect Dis. 1997 Mar;24(3):506–10. | Non-point source outbreak. |
| Yan M, Li X, Liao Q, Li F, Zhang J, Kan B. The emergence and outbreak of multidrug- resistant typhoid fever in China. Emerg Microbes Infect. 2016 Jun 22;5:e62. | Non-point source outbreak and no incubation period reported |
| Yang HH, Kilgore PE, Yang LH, Park JK, Pan YF, Kim Y, et al. An outbreak of typhoid fever, Xing-An County, People’s Republic of China, 1999: estimation of the field effectiveness of Vi polysaccharide typhoid vaccine. J Infect Dis. 2001 Jun 15;183(12):1775–80. | No exposure identified |
