# Supplementary figures and images for: Incubation period of typhoidal salmonellosis: a systematic review and meta-analysis of outbreaks and experimental studies occurring over the last century
Source: BMC Infect Dis. 2018 Sep 27;18:483. doi: 10.1186/s12879-018-3391-3 (PMC6161394; doi:10.1186/s12879-018-3391-3)

Funnel plot

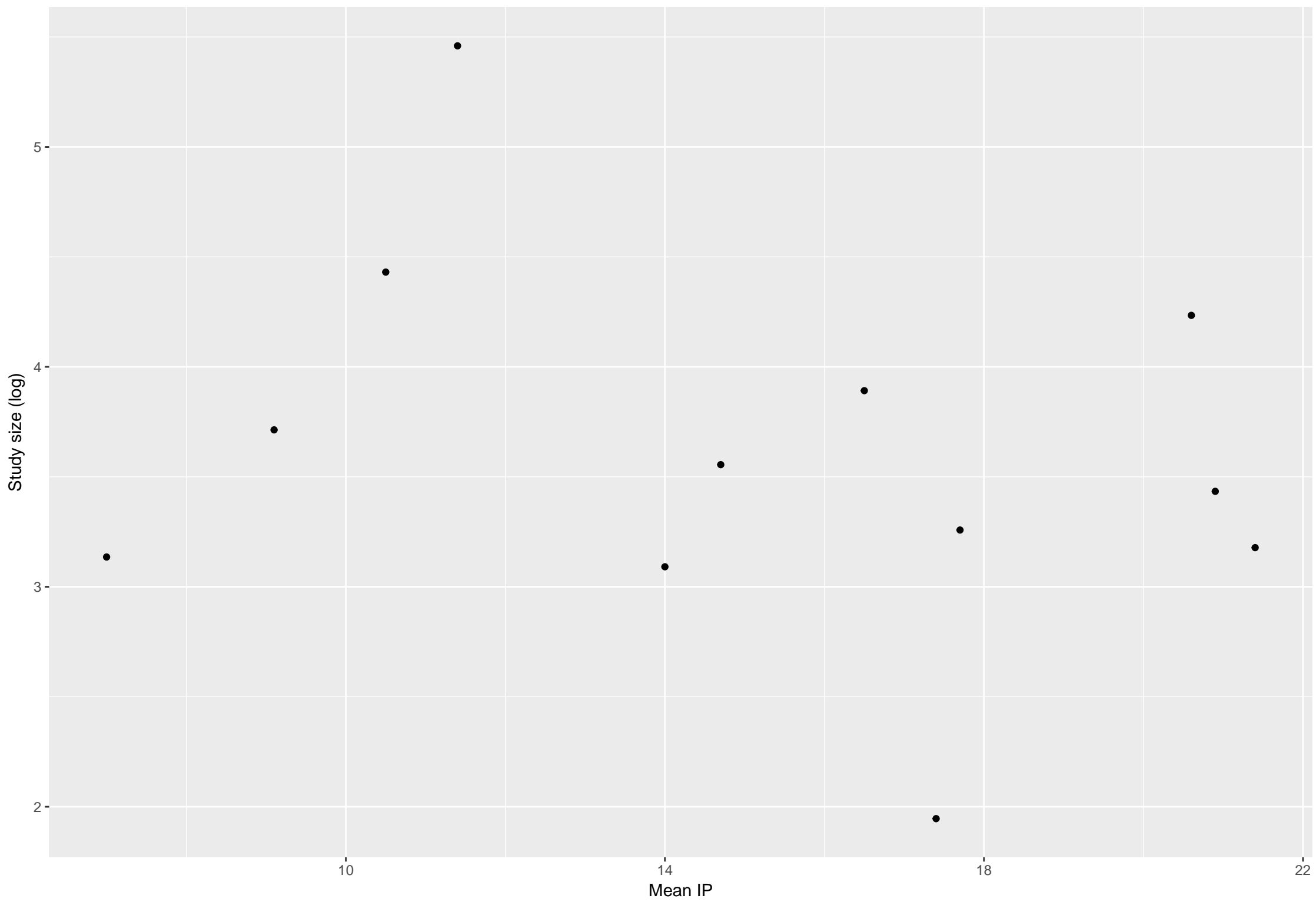

Supplement: Supplementary file 3 — Funnel plot. Graph of funnel plot showing the effect of study size on the incubation period. (PDF 5 kb) [file 12879_2018_3391_MOESM3_ESM.pdf]
